# The First New Zealanders: Patterns of Diet and Mobility Revealed through Isotope Analysis

**DOI:** 10.1371/journal.pone.0064580

**Published:** 2013-05-15

**Authors:** Rebecca L. Kinaston, Richard K. Walter, Chris Jacomb, Emma Brooks, Nancy Tayles, Sian E. Halcrow, Claudine Stirling, Malcolm Reid, Andrew R. Gray, Jean Spinks, Ben Shaw, Roger Fyfe, Hallie R. Buckley

**Affiliations:** 1 Department of Anatomy, Otago School of Medical Sciences, University of Otago, Dunedin, New Zealand; 2 Department of Anthropology and Archaeology, University of Otago, Dunedin, New Zealand; 3 Southern Pacific Archaeological Research, Department of Anthropology and Archaeology, University of Otago, Dunedin, New Zealand; 4 Community Trust of Otago Centre for Trace Element Analysis, Department of Chemistry, University of Otago, Dunedin, New Zealand; 5 Department of Chemistry, University of Otago, Dunedin, New Zealand; 6 Department of Preventative and Social Medicine, University of Otago, Dunedin, New Zealand; 7 School of Archaeology and Anthropology, College of Arts and Social Sciences, Australian National University, Canberra, Australia; 8 Canterbury Museum, Christchurch, New Zealand; New York State Museum, United States of America

## Abstract

Direct evidence of the environmental impact of human colonization and subsequent human adaptational responses to new environments is extremely rare anywhere in the world. New Zealand was the last Polynesian island group to be settled by humans, who arrived around the end of the 13th century AD. Little is known about the nature of human adaptation and mobility during the initial phase of colonization. We report the results of the isotopic analysis (carbon, nitrogen and strontium) of the oldest prehistoric skeletons discovered in New Zealand to assess diet and migration patterns. The isotope data show that the culturally distinctive burials, Group 1, had similar diets and childhood origins, supporting the assertion that this group was distinct from Group 2/3 and may have been part of the initial colonizing population at the site. The Group 2/3 individuals displayed highly variable diets and likely lived in different regions of the country before their burial at Wairau Bar, supporting the archaeological evidence that people were highly mobile in New Zealand since the initial phase of human settlement.

## Introduction

A unique aspect of living on islands is that prehistory begins with a single discrete event: colonization. However, direct evidence of human colonization events and the subsequent behavioral responses of initial settlers are rarely observable in the archaeological record. This study aims to characterize the diet of the first New Zealanders from the analysis of carbon and nitrogen stable isotope ratios in human bone collagen and to analyze human mobility through strontium isotope analysis of tooth enamel.

**Figure 1:**
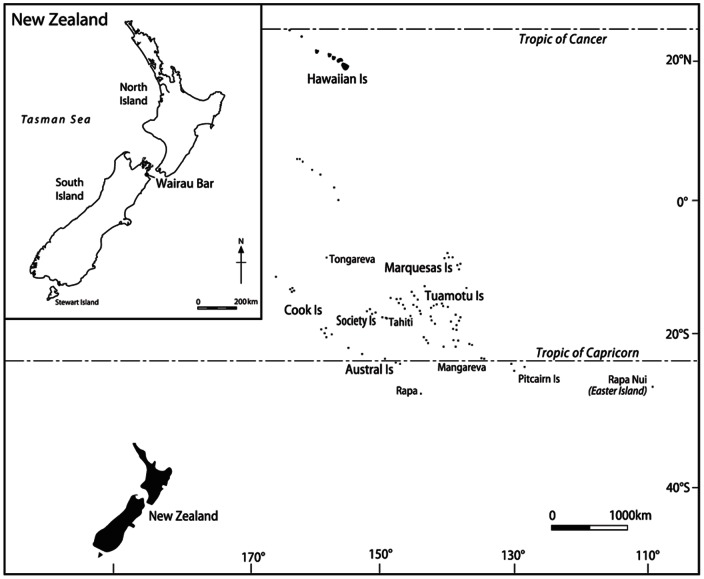
Map of Eastern Polynesia and the location of Wairau Bar, South Island New Zealand.

The Wairau Bar archaeological site, situated on the northern coast of the South Island of New Zealand (fig. 1), is the best candidate to date for a founder-phase community in which these issues may be addressed [1]. Radiocarbon dates indicate that Wairau Bar was inhabited during the earliest settlement of New Zealand, around the end of the 13th century AD [2]–[4]. Covering at least 11 ha [5], Wairau Bar is best known for its numerous burials and rich assemblage of grave goods, which include artifacts of Archaic East Polynesian (AEP) type, such as distinctive jewelry, as well as the eggs and bones of the extinct flightless moa (Aves: Dinornithiformes). Davidson et al. ([6] pg 99) have described a tool from the Wairau Bar artifact assemblage made from a shell originating in tropical waters that they argue “reinforces the view that Wairau Bar was a pioneering settlement in New Zealand”.

The exact origin(s) of New Zealand's first colonists is unknown, although all lines of evidence point to a tropical East Polynesian (TEP) homeland [7], [8]. Material culture affinities, demonstrated by the broad range of AEP artifact forms recovered from Wairau Bar, were the first line of evidence to suggest TEP origins [9]. Ancient DNA research focusing on commensal species, especially the Pacific rat (*Rattus exulans*), also indicates TEP as the likely source of the first colonists [10] and new mtDNA evidence from the Wairau Bar humans has shown that the Polynesian populations were not as genetically homogeneous as previously thought [11].

The Wairau Bar site is an important member of a group of sites of colonizing so-called ‘moa-hunters’ who moved widely in search of large game (i.e. moa and seals) and new stone resources [12]. The early colonization period of New Zealand is known for the extreme predation of fragile endemic species, most famously the moa ([3] pg 426). Recent research focused on moa aDNA suggests that all eleven species were hunted to extinction within the first 100 years of human settlement [13], [14]. Stone tool assemblages from colonization phase sites across the country provide evidence of the extremely rapid exploration and discovery of industrial resources. High levels of human mobility from the time of colonization are reflected in the long-distance movement of these resources [12]. Wairau Bar is a key example of this phenomenon, where one of the major industrial materials is obsidian sourced from Mayor Island located a 900 km sea voyage to the north [12]. High mobility during the colonizer phase of human prehistory in New Zealand is also reflected in the occupation of ‘transient villages’; sites that were likely inhabited for as little as a few decades or less [15], [16].

**Figure 2:**
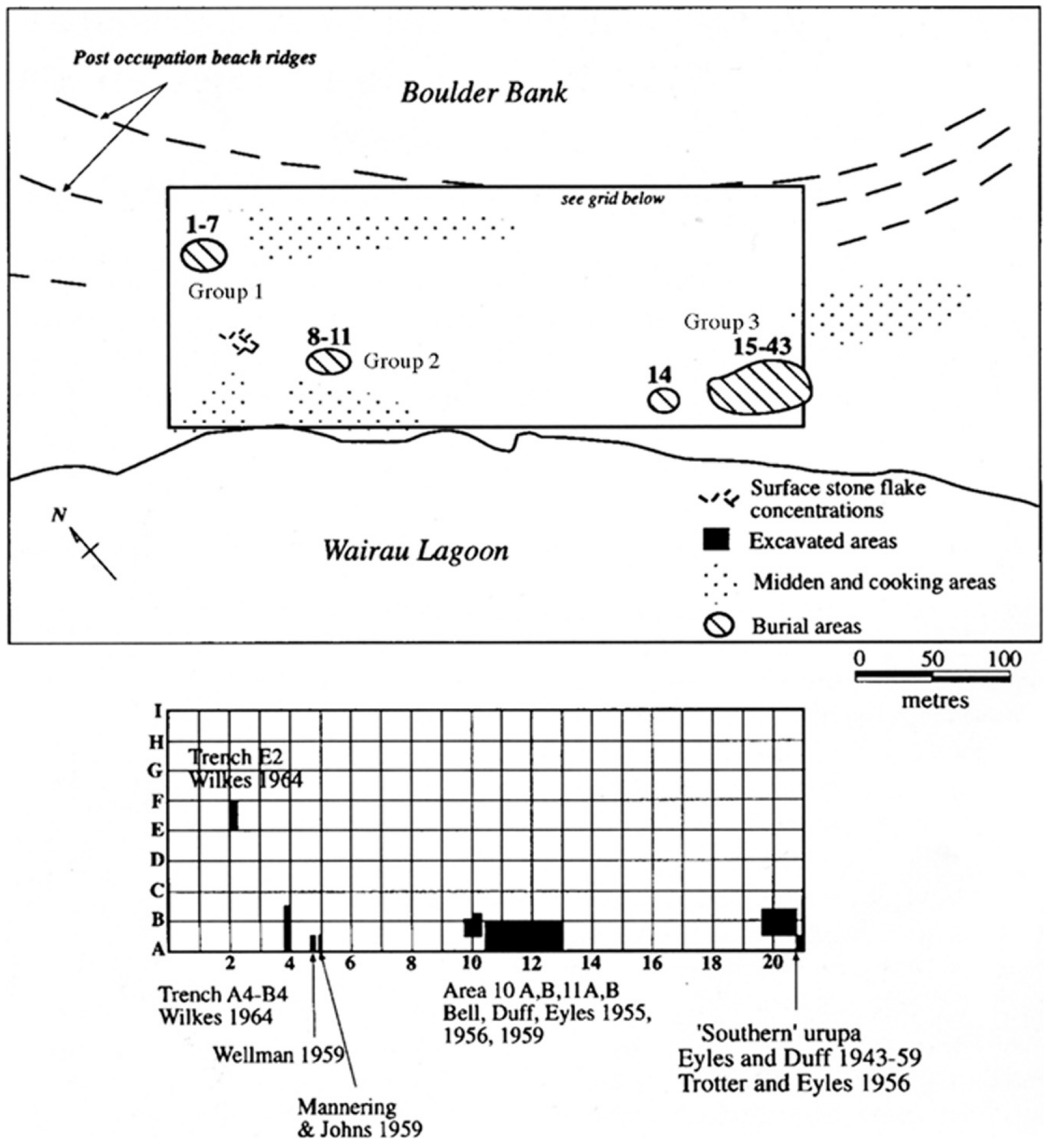
Map of the Wairau Bar site reproduced from Higham et al.[3].

The Wairau Bar burials provide the only large and well provenanced sample (n = 42) of prehistoric Maori skeletons, allowing questions of adaptation and mobility to be directly addressed through the biological remains of the people [17], [18]. The burials from the site were found in three discrete areas [19]. The first group (burials 1–7) was found in the northwest area of the site, the second group (burials 8–11) was interred in an area southeast of Group 1, and the third group comprises the remaining burials, most of which were found in what Duff [9] called ‘the southern burial ground’ (burials 12–44) (fig. 2). Artifacts found with the burials include drilled moa eggs, whale tooth and imitation whale tooth pendants, necklace reels, dolphin tooth necklaces, perforated shark teeth, minnow lures and stone adzes [19]. Although the Wairau Bar site as a whole is clearly very early and the burials all appear more or less contemporaneous (*ca.* AD 1300) on radiometric grounds [3], Group 1 has long been recognized as being distinctive by their location in the burial ground, their burial position and because grave offerings (including moa bone ornaments and moa eggs) were much more numerous in this group [1], [2], [20], [21]. Although there is some inconsistency in the numbers of grave goods found in the burials depending on the source, such as the text *vs*. tables in Duff [19] and Duff's unpublished field notebooks etc., there is a ratio of approximately 5∶1 in favor of Group 1 in terms of mean numbers of grave goods. Furthermore, moa bones were found in all but one of the Group 1 burials and all of the Group 1 individuals were interred with perforated moa eggs. In contrast, only two individuals in Group 2/3 were buried with moa bones and only six were interred with perforated moa eggs. Analysis of the skeletal and dental remains revealed differences in health and diet between Group 1 and the others [17]. Status differentiation [19], sex differentiation [20] (later refuted by [22]) and chronology [1] have each been offered as potential explanations for the differences observed between the Wairau Bar burial groups.

The carbon and nitrogen stable isotope signatures in bone collagen and strontium isotope ratios of tooth enamel are a reflection of diet and place of childhood residence, respectively [23]–[26]. Here we measure carbon, nitrogen and strontium isotope ratios in the prehistoric individuals from Wairau Bar and assess the degree to which the diet and childhood place of residence correspond with patterns of cultural differences observed between the groups. The null hypothesis tested here is that there are no isotopic differences between the groups of burials from Wairau Bar. In other words, that the burials represent a homogenous group. This would suggest that they comprise a group with a single origin and subsistence base. The alternative hypothesis is that Group 1 is significantly different from Group 2/3. This would suggest that the Groups have a different origin and diet.

### Reconstructing paleodiet and prehistoric human mobility using isotope analysis

Carbon and nitrogen stable isotope analysis of bone collagen is a well-established method of reconstructing past diets (see reviews by [23], [26], [27]). The stable isotope ratios of carbon and nitrogen from the diet are reflected, albeit slightly altered, in the body's tissues, including bones and teeth [28]. Carbon stable isotope ratios (δ^13^C) are used for dietary reconstruction because δ^13^C values differ between marine (higher values) and terrestrial (lower values) ecosystems and plants with differing photosynthetic pathways, specifically C_3_ plants (lower values) and C_4_ plants (higher values) [26], [29]. Nitrogen stable isotope ratios are used to differentiate between the consumption of plant (lower values) and animal (higher values) protein resources. The consumption of aquatic resources can also be detected using nitrogen stable isotope ratios because marine and freshwater resources typically display higher δ^15^N values compared to those from terrestrial systems [30]. Furthermore, there is an increase of ∼3–6‰ in nitrogen stable isotopes ratios and ∼0–2‰ in carbon stable isotope ratios with each trophic level [31]–[33].

Nitrogen stable isotope ratios of bone collagen only reflect the protein portion of the diet as carbohydrates and lipids do not contain nitrogen [28]. Carbon stable isotope ratios from dietary protein are preferentially ‘routed’ to bone collagen during synthesis although other dietary macronutrients may contribute carbon, especially if the overall protein intake is low [34]–[37]. Bone turnover is relatively slow and therefore the stable isotope ratios of bone collagen are representative of approximately the last 10–15 years of an adult's diet [38]. In order to interpret past diet, it is important to determine the local range of environmental isotope values of potential dietary items by analyzing faunal remains from a similar area and temporal period as the prehistoric humans.

Strontium isotopes are used to trace human mobility because the ^87^Sr/^86^Sr ratios in bone apatite and tooth enamel are primarily representative of the local geology during the time of tissue synthesis. Mineral weathering and erosion of the underlying rock, in addition to atmospheric deposition, dictate the strontium isotope ratios of the soil. Strontium isotope signatures from the leachable component of the soil are incorporated into the local biosphere from the uptake of strontium by plants. [39]. Plants are strontium rich compared to animals as a result of biopurification, the physiological discrimination against strontium in preference of calcium [24]. As a result of biopurification herbivores and carnivores assimilate less strontium than is present in the plants and animals in their diets respectively. Therefore plants provide a substantial amount of strontium to human diets compared to meat [24]. Once consumed, strontium is substituted for calcium in the hydroxyapatite of tooth enamel. As teeth do not remodel over time and are resilient to diagenetic change dental enamel is preferred over bone apatite for strontium isotope analysis [40]. Strontium isotopes have successfully been used to trace human mobility in a number of prehistoric populations around the world (e.g. [41], [42], [43]), including the Pacific islands (e.g. [44], [45]–[47]).

The ^87^Sr/^86^Sr ratio of a rock is determined by its type and age. Older rocks typically have more radiogenic strontium than more newly formed rocks because of the length of time necessary for radioactive rubidium (^87^Rb) to decay into ^87^Sr, which is then compared to a stable isotope of strontium (^86^Sr) [43]. Although ultimately derived from the underlying bedrock, ^87^Sr/^86^Sr ratios of the labile, or biologically available, strontium in local soils and water can vary for a number of reasons [48]. A mixture of rocks with differing ^87^Sr/^86^Sr ratios will contribute to the labile strontium in varying amounts depending on factors such as the rate of weathering and the type of rock [48]. Additionally, as the average ^87^Sr/^86^Sr ratio of seawater is 0.7092, sea-spray and marine-derived precipitation can potentially elevate the ^87^Sr/^86^Sr ratios of terrestrial food webs nearer to that of seawater [44]. Therefore, the analysis of the ^87^Sr/^86^Sr ratios of underlying rocks only provides a possible range for local labile ^87^Sr/^86^Sr ratios.

New Zealand is a continental island with a complex geological history spanning the last 550 million years [49]. Different regions of New Zealand were formed at various times, either from volcanism, metamorphism or sedimentation (or a mixture of these processes) from continental and oceanic crust sources interspersed with periods of massive erosion and uplift over time [50]. The West Coast and the northern part of the South Island were formed initially and therefore the oldest rocks in the country can be found in these regions. Other regions, such as Northland, Southland and Otago, were formed later by continued volcanism and/or the uplift of ocean sedimentation. Limestone sedimentary rock formed from the remains of ancient sea organisms display ^87^Sr/^86^Sr ratios representative of the prehistoric oceans during the lifespan of the animal. Continual uplift, volcanism and erosion has resulted in the mixed geology and associated broad range of strontium ratios observed throughout New Zealand [49], [51]–[59].

The islands located in the Central Pacific Basin, east of the Andesite Line, are primarily volcanic, often with extensive coral development. These volcanic and coral islands were formed relatively recently compared to continental islands such as New Zealand. They are primarily composed of basalts (^87^Sr/^86^Sr ratios between 0.702–0.704) and carbonates, such as uplifted coral, which display ^87^Sr/^86^Sr ratios close to seawater (0.707–0.709, currently 0.7092) [50]. Most tropical East Polynesian volcanic islands display geological ^87^Sr/^86^Sr ratios that are intermediate between basalts and marine-derived carbonates, although the labile strontium will be dependent on the soil composition [39], [47]. Unfortunately little research has been conducted on the ^87^Sr/^86^Sr ratios of materials other than geological samples (e.g. plants and animals) that would be more representative of the labile strontium available on tropical East Polynesian islands.

The strontium isotopic ratios of domesticated and endemic animals with small home ranges can be used as a proxy for the local labile strontium isotope signature of an area (reviewed in [48]). Variations in human strontium isotope values greater than two standard deviations (± 2SD) from the mean of the local range are then considered to be most likely ‘non-local’ to the site [39]. The use of domestic species must be used with caution as some studies have observed that animals with highly variable strontium isotope ratios were likely being brought into sites from outside areas (e.g. [45], [47]).

## Materials and Methods

Written consent for the permission to analyze the prehistoric Wairau Bar individuals was provided by the Maori descendant tribe, Rangitane ki Wairau, as part of a memorandum of understanding signed by the University of Otago, Canterbury Museum and Rangitane ki Wairau before reburial of the human remains in 2009. No permits were required for the described study, which complied with all relevant regulations. All the faunal material sampled for the current study is curated by the Department of Anthropology and Archaeology, University of Otago, Dunedin, New Zealand. Bone was sampled from thirty-eight human individuals and eighty-eight prehistoric animals for carbon and nitrogen stable isotope analysis. When possible cortical bone was sampled from long bones, although other bones were chosen if these were not available.

The method used to extract the collagen from all bone samples followed the procedures of Brown et al. [60] and Collins and Galley [61]. Specifically chunks of bone weighing ∼1.0–1.5 g were cleaned with alum oxide air abrasive equipment (Bego Easyblast), demineralized in 0.5 M HCl at 4°C for several days followed by rinsing in de-ionized H_2_O until the samples reached a neutral pH. The samples were gelatanized at 70°C in a pH 3 solution for 48 hours, followed by filtering with 5–8 μm Ezee® mesh filters (Elkay Laboratory Products) to remove any reflux-insoluble residues and ultrafiltered with Millipore Amicon Ultra-4 centrifugal filters (30,000 NMWL) to retain molecules larger than 30 kDa. The purified “collagen” was lyophilized for 48 hours and analyzed by EA-IRMS (Finnigan Mat 252 differentially pumped isotope ratio mass spectrometer) coupled with a Europa Scientific elemental analyzer at Iso-Analytical (Cheshire, UK). Stable isotope ratios are expressed relative to international standards (VPDB for carbon and AIR for nitrogen) by means of the delta (δ) notation in parts per thousand, or per mil (‰). All samples were run in duplicate. An internal standard, IA-R042, was used as reference material to ensure the analytical precision of the measurements for the sample analyses. IA-R042, a mixture of IA-R005 and IA-R045 and a mixture of IA-R006 and IA-R046 were analyzed for quality control of the samples. Analytical error was routinely ±0.1‰ for δ^13^C, ±0.2‰ for δ^15^N. Samples that did not fall within the collagen quality criteria of a C/N ratio of 2.9–3.6 [28] were removed from statistical analyses and interpretations.

Twenty-four human teeth and five dogs' teeth were sampled for strontium isotope analysis. All human teeth samples were early forming teeth (<7 years old) except for three burials (burials 5, 21 and 30), which were third molars that finish development during the teenage years. Strontium was purified and analyzed at the Community Trust of Otago Centre for Trace Element Analysis at the University of Otago [62] as outlined in Shaw et al. [47]. Tooth samples were ultrasonicated in ultra clean Milli-Q water (18.2 MOhm.cm resistivity @ 25°C, <5 ppb Total Organic Carbon (TOC)) and methanol. Following this step, a small sliver (5–50 mg) of enamel was sawed off with a Dremel® diamond cutting wheel. The enamel surface and any remaining dentine were removed with a tungsten carbide drill bit. Chemical purification of the enamel strontium was performed in a class 10 clean room. Enamel was leached in cold 4 M HCl for ∼5 minutes, rinsed multiple times in Milli-Q water, and digested in 3 M HNO_3_. Strontium was eluted and purified from the samples using a micro-chromatographic exchange column loaded with Eichrom Sr-SPEC resin (Eichrom Technologies, U.S.A.) following a slightly modified version of the method outlined in Pin and Bassin [63]. Strontium isotope ratios were measured with a Nu Plasma MC-ICP-MS parallel to the strontium isotope standard SRM 987. The analytical error (±1 SE) for the strontium analyses ranged from 0.000009–0.000015.

## Results

### Paleodiet at Wairau Bar

#### Animal bone data

**Figure 3:**
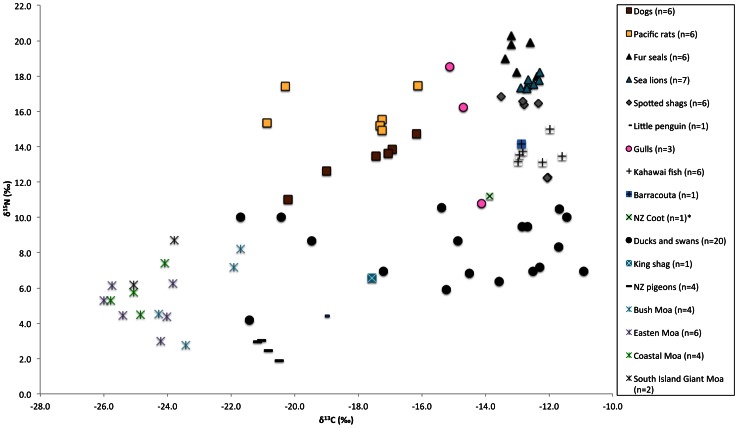
Wairau Bar faunal bone collagen δ^13^C and δ^15^N values.

Stable isotope analysis of carbon and nitrogen was conducted on bone collagen from twenty-three animal species from a Wairau Bar midden to provide environmental isotopic ranges of the potential dietary items at the site (fig. 3). The site has no evidence for stratification, which implies that it represents a single-phase occupation. All radiocarbon dates agree that this occupation most likely occurred during the late 13^th^ and early 14^th^ centuries AD and thus the midden and burials must be considered broadly contemporaneous [3]. The carbon and nitrogen stable isotope ratios of eighty-six prehistoric animals from Wairau Bar yielded good quality collagen (Table S1). Five of the species of avifauna analysed are now extinct, including four moa species (*Emeus crassus*, *Anomalopteryx didiformis*, *Euryapteryx curtus*, *Dinornis robustus*) and one species of rail (*Fulica prisca*).

The δ^13^C and δ^15^N values of marine mammals averaged (± 1SD) −12.7‰±0.4‰ and 18.4‰±1.0‰, respectively and indicate that these animals fed on open water and pelagic species of fish. The frugivorous New Zealand pigeon (*Hemiphaga novaeseelandiae*) δ^13^C and δ^15^N values averaged −20.9‰±0.3‰ and 2.6‰±0.5‰ respectively. The low δ^13^C and higher δ^15^N values of the moa species (−24.3‰±1.3‰ and 5.6‰±1.7‰) compared to the pigeons may indicate the consumption of plants and seeds depleted in ^13^C and enriched in ^15^N by moa. The low δ^13^C values of the moa may be explained by these species browsing ^13^C-depleted plants from the rainforest floor as a result of the ‘canopy effect’ [64]. Species of duck and swan display stable isotope values (−15.0‰±3.6‰ and 8.2‰±1.8‰ for δ^13^C and δ^15^N respectively) indicating that low trophic level fish, invertebrates and aquatic plants from marine, freshwater and brackish environments, likely the lagoon, estuary and river near Wairau Bar, were primary food sources. The δ^13^C and δ^15^N values of the marine birds (−13.9‰±2.4‰ and 13.0‰±4.5‰) and gulls (−14.6‰±0.5‰ and 15.2‰±4.0‰) indicate these species consumed foods from higher trophic levels, such as pelagic species of fish, compared to the ducks and swans. The dogs and rats displayed δ^13^C and δ^15^N values (−17.8‰±1.5‰ and 13.2‰±1.3‰ and −18.2‰±1.9‰ and 16.0‰±1.1‰ respectively) indicative of a mixed marine and terrestrial diet from higher trophic levels, likely reflecting the values of human food scraps at the site.

#### Prehistoric human diet

**Figure 4:**
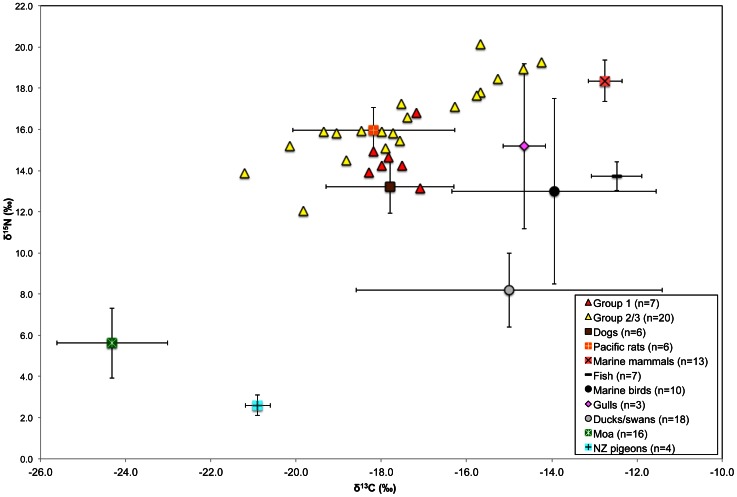
Wairau Bar human bone collagen δ^13^C and δ^15^N values with reference to the dietary baseline.

Demographic data, burial information and isotope values for the humans are located in Table S2. Eleven individuals from Group 2/3 did not display good quality collagen and were excluded from the following statistical analyses and interpretations. Although the average δ^13^C values were similar (0.2‰ difference), the average δ^15^N value of the Group 1 (n = 7) individuals was statistically significantly lower (1.9‰, Student's t-test *p* = 0.025) than the Group 2/3 (n = 20) individuals, indicating the consumption of lower trophic level protein resources by the Group 1 individuals. Although the sample size was small for multivariate modeling, the difference in δ^15^N values between the groups was not found after adjusting for sex (*p* = 0.150) and therefore the unadjusted finding should be interpreted with some caution. Importantly, the variability in δ^13^C values (but not δ^15^N values) was significantly different between Group 1 and Group 2/3 (Levene's test *p* = 0.014), suggesting that the diet of the latter was much more variable (fig. 4).

Group 1 individuals displayed similar δ^13^C and δ^15^N values (−17.7‰±0.5‰ and 14.6‰±1.1‰ respectively), which indicates that all these individuals consumed a comparable diet within a span of 10–20 years prior to their death. If the members of Group 1 had access to marine foods including fish, shellfish and lower trophic level terrestrial foods, a positive correlation would be expected between the δ^13^C and δ^15^N values, and this is not the case. Therefore, the lack of any correlation between δ^13^C and δ^15^N values (Spearman's *r* = 0.00, *p* = 1.000) indicates the protein resources for Group 1 were either marine and terrestrial resources from similar trophic levels *or* consisted of one major protein resource, such as domesticated animals with controlled diets from these two ecosystems.

A stable isotope study of prehistoric individuals from the Hanamiai site in the Marquesas Islands, French Polynesia, identified a similar dietary trend to that observed in the Group 1 burials. The Hanamiai individuals displayed high δ^15^N and δ^13^C values indicating the consumption of pigs (*Sus scrofa*) and other domestic animals such as dogs (*Canis familiaris*) and rats (*Rattus exulans*) that consumed both marine and terrestrial resources [65]. In Polynesia, low protein starchy vegetables such as taro, sweet potato and breadfruit comprise a large proportion of the diet [66], but the low-protein nature of these foods may render them less visible isotopically than higher protein meat products [28]. If the individuals in Group 1 were among the initial founders of Wairau Bar from a TEP homeland and they had died before a complete turnover of bone had occurred (10–20 years), the stable isotope values of their bone collagen would mostly represent their diet before coming to New Zealand. The carbon and nitrogen stable isotope ratios of Group 1 indicate a diet with a low diversity in protein resources. This may be representative of the TEP-like diet consisting of protein primarily derived from domestic species, similar to the pattern found by Richards et al. [65].

Another possible explanation for the δ^13^C and δ^15^N values of the Group 1 individuals may be the consumption of marine and terrestrial protein resources that displayed similar δ^15^N values, such as marine shellfish [67], wetland bird species and terrestrial birds, for a number of years before death. This dietary pattern may be representative of hunting and gathering around Wairau Bar or other areas of New Zealand before interment at the site. However, the lack of higher trophic level marine foods, especially marine mammals, is not consistent with the archaeological evidence at numerous early sites in New Zealand, especially in the South Island (including Wairau Bar). The high concentration of marine mammal remains at these sites indicate these taxa were highly sought after since the earliest phases of human occupation [8], [68]–[71].

The Group 2/3 δ^13^C and δ^15^N values were considerably more variable and ranged from −21.2‰ to −14.2‰ for δ^13^C values and 12.1‰ to 20.2‰ for δ^15^N values (mean ± 1 SD for δ^13^C and δ^15^N values were −17.5‰±1.9‰ and 16.4‰±1.9‰ respectively). This extreme variation in δ^13^C values within Group 2/3 reveals substantial intra-group dietary variation between marine and terrestrial ecosystems. A significant positive correlation of δ^13^C and δ^15^N values (Spearman's *r* = 0.89, *p*<0.001) indicate the protein portion of their diet consisted of high trophic level marine foods (sea mammals, marine birds and pelagic/carnivorous species of fish) and lower trophic level terrestrial foods (likely endemic bird species) [72]. Even if a large trophic level enrichment for carbon (+2‰) and nitrogen (+6‰) between predator and prey bone collagen is applied, it is obvious that marine foods contributed a substantial amount of protein to the Group 2/3 diets. However, it is difficult to clearly differentiate between marine birds and seals, as they both display high δ^13^C and δ^15^N values. Higher trophic level fish from pelagic zones may also have contributed to the marine protein resources of Group 2/3. Compared with the moa, the human Group 2/3 δ^15^N values were elevated by 10.8‰ and δ^13^C values by 6.8‰. The difference between the δ^13^C and δ^15^N values of humans and smaller birds was larger: 3.3‰ and 13.9‰ for the New Zealand pigeons (*Hemiphaga novaeseelandiae*) and −2.5‰ and 8.3‰ for the ducks and swans. Birds from wetland areas were also likely consumed, as observed from the archaeological avifauna remains at the site [73], but were not as important as higher trophic level marine animals.

These isotopic data also indicate that dog and rat may have been eaten by Group 2/3 and, correspondingly, the diets of these animals suggest they fed off human food scraps. The high δ^15^N values of the humans from Group 2/3 with lower δ^13^C values may also indicate the consumption of freshwater organisms. It is possible that animals such as eel (*Anguilla* sp.), a species documented as a food resource later in the historic period elsewhere in New Zealand [74], were also exploited but baseline data are not currently available to support this.

The dietary pattern observed in Group 2/3 fits the prediction of a population exploiting a wide range of protein resources accessible during the earliest stages of human settlement. The large variation in stable isotope values within Group 2/3 had no relationship with age or sex. In this group there is a wide spectrum of possible food sources ranging from primarily high trophic level marine species for individuals at one end, to terrestrial resources in those at the other end, with a mixed diet of marine foods with terrestrial resources in the middle.

Archaeological evidence supports an early emphasis on large meat animals such as moa and sea mammals [68]–[70], [75]. However, fur seals (*Arctocephalus forsteri*) were probably the most important source of meat for the first generations of settlers except in places where there were very high densities of moa and poor hauling-out zones for seals [8], [71]. This includes the east coast of the South Island between Dunedin and Banks Peninsula (far south of Wairau Bar), which is the only zone where moa has been shown to have provided a greater contribution to diet than seals [8].

Early period settlements in New Zealand are thought to be ‘transient villages’ occupied for brief periods (possibly less than 20 years), which were accompanied by short-duration specialist sites for hunting, gathering, and stone quarrying activities [15], [16]. The variable dietary pattern observed in Group 2/3 supports this model and may indicate people were highly mobile before their interment at Wairau Bar. These stable isotope data also support the proposal that the Group 1 individuals were not at Wairau Bar long enough for their isotope values to reflect the expected broad spectrum colonizer-type subsistence hypothesized for the earliest settlers of New Zealand.

### Prehistoric human mobility at Wairau Bar

The analysis of archaeological animal tooth enamel to characterize the ‘local’ biologically available strontium isotope signature is recommended for prehistoric mobility studies [39]. Therefore, strontium isotope analysis was conducted on five prehistoric dogs' teeth from the Wairau Bar site to establish an estimate of the range of biologically available strontium isotope ratios for the Wairau Valley. The ^87^Sr/^86^Sr ratios of the dogs were tightly clustered (0.7089±0.0001) and were well within the wide range of the local Marlborough geological ^87^Sr/^86^Sr ratios (0.7041 to 0.7308). The dog ratios fell between the range of geological ^87^Sr/^86^Sr ratios for the Wairau region and seawater (0.7092) and therefore may have been influenced by precipitation of a marine origin or sea-spray [55], [59]. Dogs are primarily carnivorous and therefore the consumption of marine foods may have also influenced the dog enamel ^87^Sr/^86^Sr ratios [44]. However, the stable isotope evidence indicates that dogs from the site were also consuming terrestrial foods, which would be representative of the local labile strontium signature. The Wairau dogs' ^87^Sr/^86^Sr ratios display a similar standard deviation (±0.0001) to pigs used by Bentley et al. [76] to define the ‘local’ labile strontium signature at a prehistoric site in Germany. The tight clustering of the dog ^87^Sr/^86^Sr ratios supports their local origin and, accordingly, they are used as a proxy for the local labile strontium baseline of the Wairau region.

**Figure 5:**
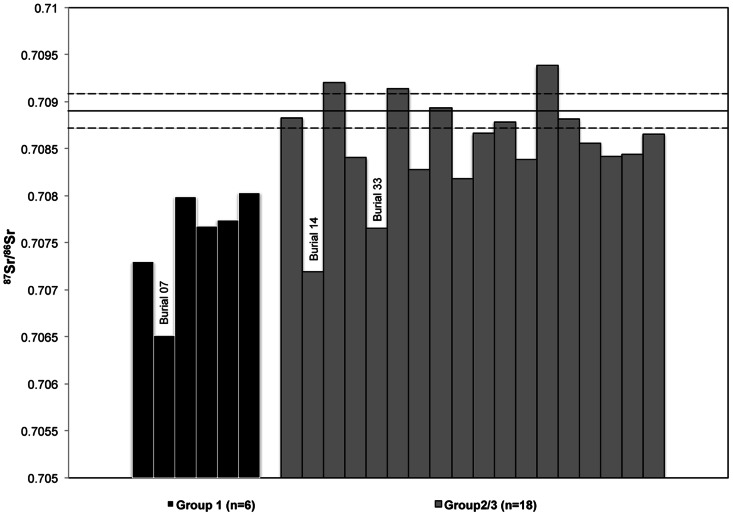
Human ^87^Sr/^86^Sr ratios compared to mean dog ^87^Sr/^86^Sr ratio (solid line) ±2 SD (dotted lines).

The six individuals from Group 1 had an average ^87^Sr/^86^Sr ratio of 0.7075±0.0006, 0.0014 lower (*p*<0.001) than the mean dog ^87^Sr/^86^Sr ratio. Group 2–3 had an average ^87^Sr/^86^Sr ratio of 0.7086±0.0005, 0.0003 lower than the mean dog ^87^Sr/^86^Sr ratio, with no evidence of a difference between the dogs and Group 2/3 (*p* = 0.132) (fig. 5).

Regression models were used to compare the ^87^Sr/^86^Sr ratios of the groups both with and without adjustment for possible sex differences (overall difference between groups *p*<0.01 in both cases). These models indicated that there was a significant difference in ^87^Sr/^86^Sr ratios between Group 1 and Group 2/3 without adjustment for sex (0.0010 higher in Group 2–3, *p* = 0.001) and with adjustment for sex, 0.0011 higher in Group 2–3, *p* = 0.006). This difference between Group 1 and Group 2/3 strongly suggests these individuals spent their childhood in geologically different areas.

Given that the strontium results from Group 2/3 are closer to those of the local dog sample than to Group 1, a reasonable interpretation of this pattern is that the Group 1 individuals were immigrants to the site while some of the Group 2/3 individuals had resided in or near the Wairau Bar region during childhood. Two males from Group 2/3 (Burial 14 and Burial 33) displayed ^87^Sr/^86^Sr ratios suggesting that they also came into the Wairau region from an area with similar labile ^87^Sr/^86^Sr ratios to Group 1.

Without more baseline data for the biologically available strontium isotope signatures of the Marlborough region and surrounding areas, the large range in Group 2/3 ^87^Sr/^86^Sr ratios, many of which are greater than two standard deviations from the average dog ^87^Sr/^86^Sr ratios, could be interpreted in a number of ways. The ^87^Sr/^86^Sr ratios may indicate that many of the Group 2/3 individuals spent their childhood in regions outside of Wairau Bar, but areas still geologically different from Group 1. A wide geographical range used for hunting and gathering may also account for the variation in Group 2/3 ^87^Sr/^86^Sr ratios, as has been suggested by another strontium isotope study of prehistoric people from Ban Chiang, Thailand [77]. This would be especially true if wild plants, such as fern root and cabbage tree (*Cordyline australis*), or horticultural species such as sweet potato (*Ipomea batatas*), were being harvested or transported from different regions. As plants contribute substantially more strontium to the body than meat it is possible the carnivorous dogs were eating animals caught locally, as suggested by Scofield et al. [73] from the distribution of bird species, but the human plant foods came from farther afield. It must be noted that although sweet potato (*kumara*) was brought with the initial colonists from TEP and can be grown in areas north of the Banks Peninsula, it would have been used only as a supplement to hunting and gathering subsistence economies outside the optimal horticultural zones in the North Island [16].

Because of the overlap in Pacific island bedrock ^87^Sr/^86^Sr ratios, the strontium isotope ratios of the Group 1 individuals (and possibly Burials 14 and 33 from Group 2/3) could represent a wide variety of potential bedrock sources within New Zealand and abroad, including a mixture of basalt and limestone typical of Oceanic islands in TEP and the North Island of New Zealand (see supporting online material, figs. S1 and S2). The mixing of marine-derived precipitation and sea-spray on small TEP islands and coastal New Zealand areas may also have influenced labile ^87^Sr/^86^Sr ratios closer to that of sea-water (0.7092), as has been shown for islands in the UK [78], [79]. However, as Group 1 exhibits the lowest values of all the individuals it is likely they have not been significantly affected by marine-derived strontium. Although some individuals may be affected by marine-derived strontium in Group 2/3, the variation and range of ^87^Sr/^86^Sr ratios in this group still suggest that they likely came from different regions.

**Figure 6:**
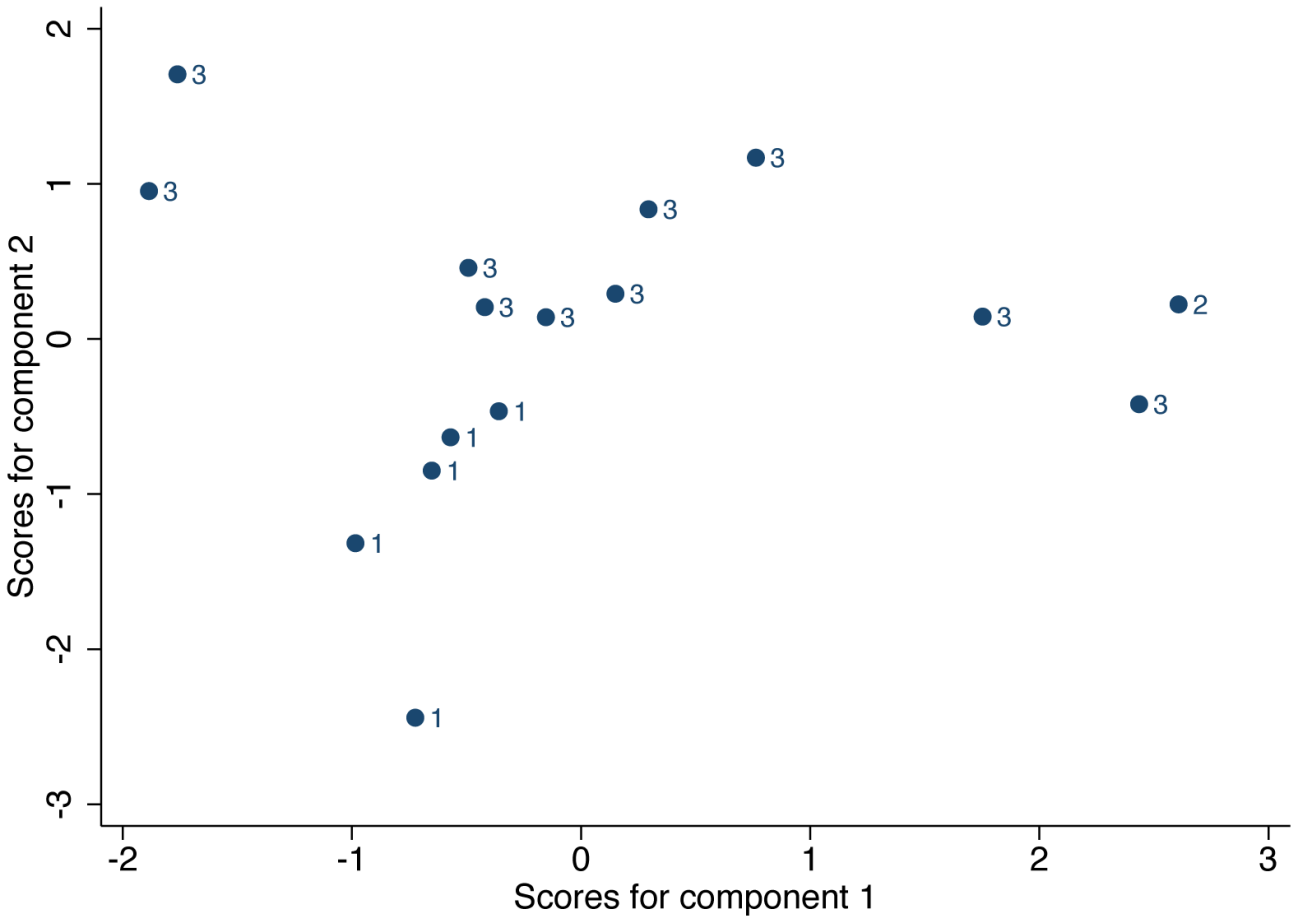
Principal components analysis of the human isotope data (δ^13^C, δ^15^N. **and ^87^Sr/^86^Sr ratios).**

A principal components analysis was used to examine the carbon and nitrogen stable isotope results and the strontium isotope ratios of the Wairau Bar individuals. Two principal components were sufficient to explain 58% and 35% respectively (total 92%) of the variance in the data. Component one reflected high carbon and nitrogen values while component two reflected high strontium values with a moderate negative carbon loading. Figure 6 shows a scatterplot of the two component scores with points labeled by their group. Individuals from Group 1 are clearly separable from those in Group 2/3 (even using only the second component representing mainly strontium values) and one individual from Group 2 is at the edge of this cluster.

## Discussion and Conclusions

This study clearly allows the null hypothesis, that there will be no difference between the groups, to be rejected. The alternative hypothesis, that the isotope data will show Group 1 is significantly different from Group 2/3 is not falsified. While these data cannot identify a specific origin for the individuals from Group 1 (and the two males from Group 2/3) it would seem that the Wairau Bar community was composed of individuals from different origins and it is possible that the Group 1 individuals were part of the founding group. Possible support for this interpretation is found in the investigation of existing archaeological data from the site, as outlined in the following section.

As Pollard ([25] pg 637) states “it is important to consider first and foremost the isotope data in a wider archaeological context, and be prepared to look for something which has changed or perhaps, in this case, something which is different rather than looking *ab initio* for a specific postcode of origin. A key test is to ask whether the rest of the archaeological evidence supports or refutes such observations”. With this in mind, the analysis of the isotope data demonstrates the distinctiveness of Group 1 within the Wairau Bar. Because human communities can be influenced by non-biological factors, it is important to consider whether Group 1 was also distinctive in other respects.

Higham et al. [3] have suggested that the scarcity of moa bone ornaments in the Group 2/3 graves could be a reflection of decreasing moa populations as a result of human predation, although there was no radiocarbon evidence of a chronological difference between Group 1 and the rest of the burials. Therefore, if the presence of moa remains in the graves is any indication of time-depth at the site, then the Group 1 burials may be the earliest. Indeed, current radiometric methods are incapable of distinguishing between two sets of ‘events’ at Wairau Bar (the groups of burials) that may have been separated by only a generation or so. The presence of numerous AEP-type artifacts and moa remains (including complete eggs) in the graves of Group 1, in addition to the similarity in grave location and burial type of the Group 1 individuals, suggests a shared cultural identity that is not evident in the rest of the cemetery. The preference for a prone burial position for all of the Group 1 individuals, but variable burial positions for Group 2/3, also supports the concept of ritual continuity with a parent community that did not continue beyond the first generation after initial settlement.

The archaeological evidence, including the fact that Wairau Bar is one of the earliest sites in New Zealand, combined with the isotope evidence for a geologically similar origin and similar diet, suggests Group 1 may represent a founding population. While the strontium data cannot identify a specific origin for the individuals from Group 1 (and the two males from Group 2/3) it would seem that the Wairau Bar community was comprised of individuals from different origins with different diets. By integrating the stable carbon and nitrogen isotope data with the strontium isotope ratios, the variability of diet in Group 2/3 likely supports the concept of high human mobility hypothesized by Walter et al. [12] during the colonizer phase of New Zealand prehistory. However, the similarity of diets between the Group 1 individuals indicates a less diverse subsistence base for a period of at least 10 years before death. A low diversity in diet is difficult to imagine in the earliest phases of New Zealand prehistory, which is known for its abundance of endemic marine and terrestrial species [8]. Dental health assessments further support the suggestion of dietary differences between the groups [17]. Group 1 displayed high rates of severe periodontal disease and less severe tooth wear (especially in the anterior teeth) compared to the Group 2/3 individuals [17]. The higher rates of tooth wear in Group 2/3 may be a result of a transition to more fibrous wild plant foods over time [80], [81].

Wairau Bar stands apart from all other sites of the Polynesian colonization phase because of the combination of its size, its number of burials and the range of activities represented in the material culture and structural remains at the site [5]. As well as burials, Wairau Bar has the full complement of features such as earth ovens, middens, artifact production zones, and post-hole evidence of structures that would be expected in a permanent village. This raises the question of what sort of village this was. This complement of features and the large number of burials may indicate that Wairau Bar was a ‘central place’ during the early settlement of New Zealand, perhaps fulfilling both a ceremonial and residential (home-base) function. If, as seems likely, it was a ‘central place’ for a highly mobile founder population, then it is not unreasonable for an individual's body to have been returned to the site for interment. Indeed, this may be seen as an early manifestation of the *tangihanga* ritual which involves the body being interred in ancestral lands [82], [83] and is important to this day.

This study has offered some exciting preliminary findings about New Zealand's early prehistory and has also highlighted the lack of comparative baseline data in the region. Further characterization of the biologically available strontium in large early sites from New Zealand and TEP will be a useful future avenue of research. However, these data have shed light on the lifestyles of the people involved in an initial colonization event and the transformation from a tropical Polynesian way of life to becoming the first New Zealanders.

## Supporting Information

Figure S1 ^87^Sr/^86^Sr ratios of multiple regions of New Zealand, green shaded area depicts the Marlborough region and the red dot delineates Wairau Bar.
(TIF)

Figure S2 ^87^Sr/^86^Sr ratios of selected South Pacific islands compiled from various studies and reproduced from Shaw et al. [47].
(TIF)

Table S1 Sampling information and isotope data for the Wairau Bar fauna.
(DOCX)

Table S2 Demographic information, burial specifics, and isotope data for the Wairau Bar humans.
(DOCX)
