# Correction: The First New Zealanders: Patterns of Diet and Mobility Revealed through Isotope Analysis

**DOI:** 10.1371/annotation/05b5437f-cea1-43f4-88c1-53f1377683ef

**Published:** 2013-09-10

**Authors:** Rebecca L. Kinaston, Richard K. Walter, Chris Jacomb, Emma Brooks, Nancy Tayles, Sian E. Halcrow, Claudine Stirling, Malcolm Reid, Andrew R. Gray, Jean Spinks, Ben Shaw, Roger Fyfe, Hallie R. Buckley

In the Author Contributions statement, the last author (HRB) was incorrectly omitted from the list of those authors who wrote the paper. 

The Author Contributions statement should read: "Conceived and designed the experiments: RLK HRB BS. Performed the experiments: RLK CS MR BS. Analyzed the data: RLK AG. Contributed reagents/materials/analysis tools: CS MR JS RF RKW. Wrote the paper: RLK RKW CJ EB NT SEH HRB." 

