# Supplementary figures and images for: The First New Zealanders: Patterns of Diet and Mobility Revealed through Isotope Analysis
Source: PLoS One. 2013 May 15;8(5):e64580. doi: 10.1371/journal.pone.0064580 (PMC3654917; doi:10.1371/journal.pone.0064580)

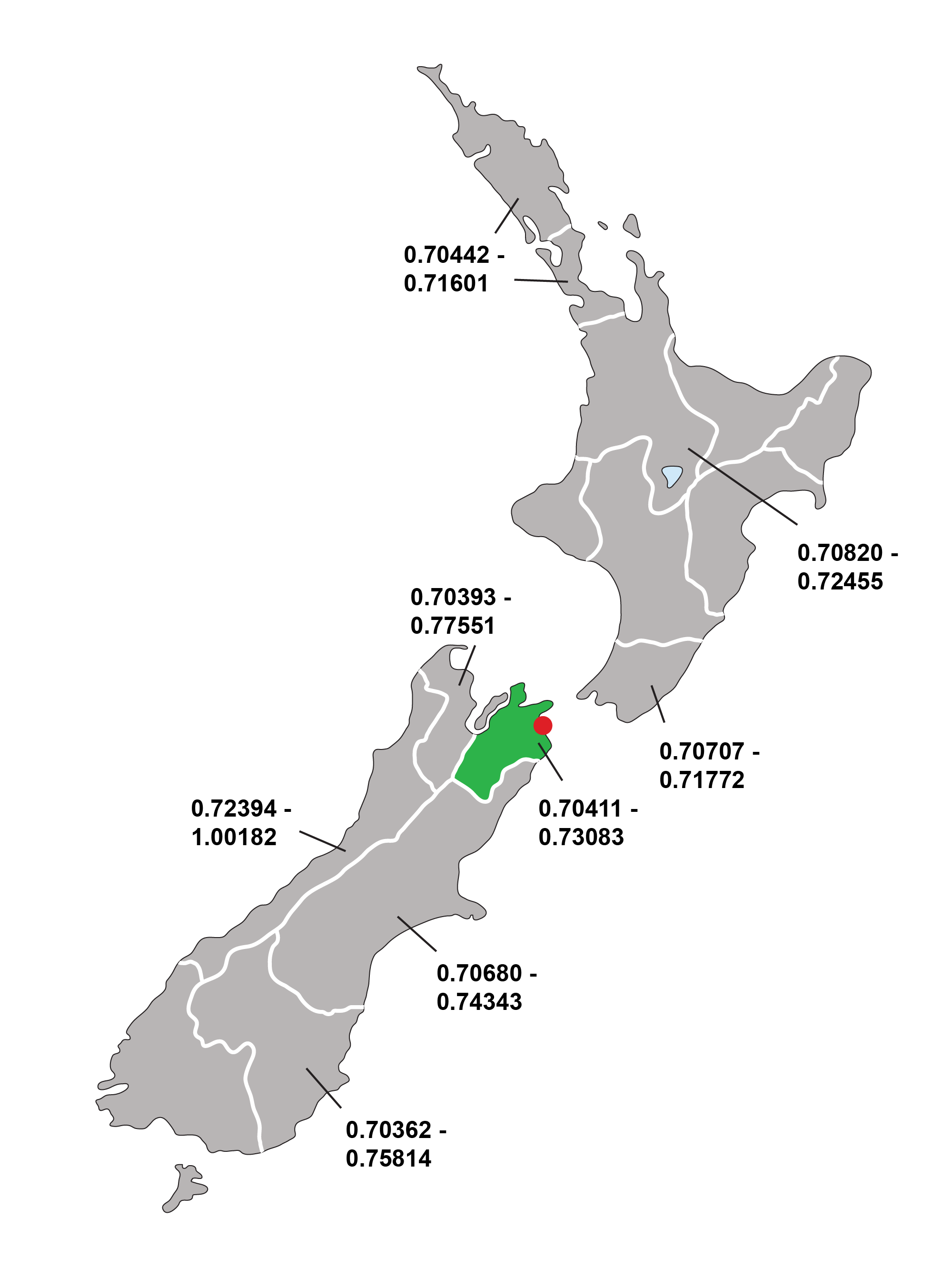

Supplement: Figure S1 — 87Sr/86Sr ratios of multiple regions of New Zealand, green shaded area depicts the Marlborough region and the red dot delineates Wairau Bar. (TIF) [file pone.0064580.s001.tif]

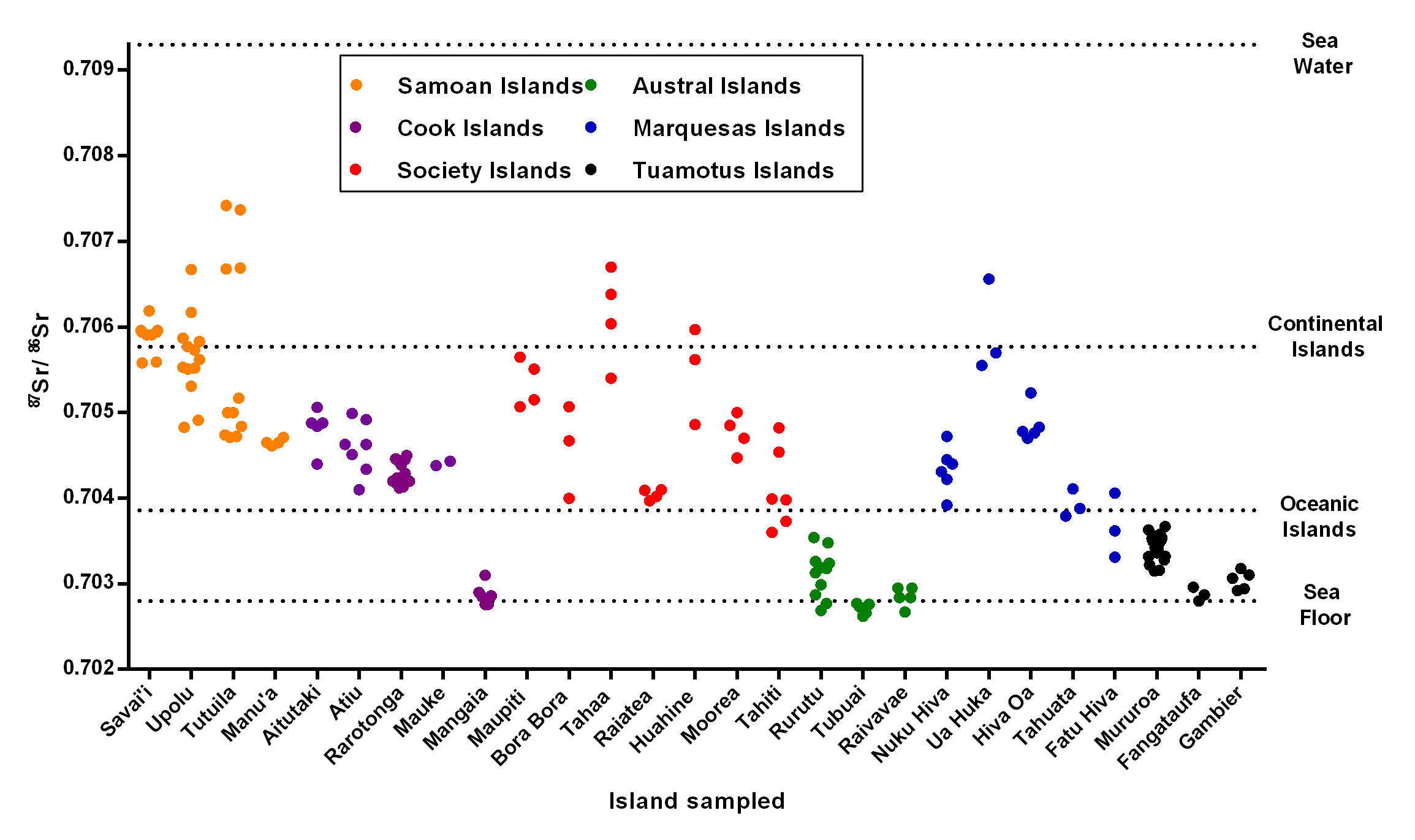

Supplement: Figure S2 — 87Sr/86Sr ratios of selected South Pacific islands compiled from various studies and reproduced from Shaw et al. [47]. (TIF) [file pone.0064580.s002.tif]
