# Supplementary material for: The First New Zealanders: Patterns of Diet and Mobility Revealed through Isotope Analysis
Source: PLoS One. 2013 May 15;8(5):e64580. doi: 10.1371/journal.pone.0064580 (PMC3654917; doi:10.1371/journal.pone.0064580)
Supplement: Table S1 — Sampling information and isotope data for the Wairau Bar fauna. (DOCX) [file pone.0064580.s003.docx]

Table S1. Sampling information and isotope data for the Wairau Bar fauna.

| Lab ID | Scientific name | Common name | Element sampled | ^87^Sr/^86^Sr | ±^87^Sr/^86^Sr  (2 S.E.) | %N | δ^15^N (‰) | %C | δ^13^C (‰) | C:N ratio |
| --- | --- | --- | --- | --- | --- | --- | --- | --- | --- | --- |
| WB dog 1 | *Canis familiaris* | dog | canine | 0.708890 | 0.000011 |  |  |  |  |  |
| WB dog 2 | *Canis familiaris* | dog | canine | 0.708790 | 0.000012 |  |  |  |  |  |
| WB dog 3 | *Canis familiaris* | dog | canine | 0.708997 | 0.000013 |  |  |  |  |  |
| WB dog 4 | *Canis familiaris* | dog | canine | 0.708843 | 0.000012 |  |  |  |  |  |
| WB dog 5 | *Canis familiaris* | dog | canine | 0.708990 | 0.000012 |  |  |  |  |  |
| WF1 | *Canis familiaris* | dog | humerus |  |  | 10.7 | 11.0 | 30.8 | -20.2 | 3.4 |
| WF2 | *Canis familiaris* | dog | humerus |  |  | 13.9 | 14.7 | 38.5 | -16.2 | 3.2 |
| WF3 | *Canis familiaris* | dog | humerus |  |  | 9.8 | 12.6 | 29.1 | -19.0 | 3.5 |
| WF4 | *Canis familiaris* | dog | humerus |  |  | 13.7 | 13.8 | 38.7 | -16.9 | 3.3 |
| WF5 | *Canis familiaris* | dog | humerus |  |  | 14.6 | 13.6 | 41.1 | -17.1 | 3.3 |
| WF6 | *Canis familiaris* | dog | humerus |  |  | 14.5 | 13.5 | 40.9 | -17.4 | 3.3 |
| WF7 | *Arctocephalus forsteri* | fur seal | humerus |  |  | 8.2 | 18.2 | 25.6 | -13.0 | 3.6 |
| WF8 | *Arctocephalus forsteri* | fur seal | ulna |  |  | 12.7 | 19.0 | 36.2 | -13.4 | 3.3 |
| WF9 | *Arctocephalus forsteri* | fur seal | humerus |  |  | 11.5 | 18.0 | 33.0 | -12.4 | 3.3 |
| WF10 | *Arctocephalus forsteri* | fur seal | radius |  |  | 14.7 | 19.9 | 42.3 | -12.6 | 3.4 |
| WF11 | *Arctocephalus forsteri* | fur seal | ulna |  |  | 13.8 | 19.8 | 39.1 | -13.2 | 3.3 |
| WF12 | *Arctocephalus forsteri* | fur seal | ulna |  |  | 12.5 | 20.3 | 35.6 | -13.2 | 3.3 |
| WF13 | *Phocarctos hookeri* | sea lion | humerus |  |  | 10.1 | 17.3 | 29.7 | -12.9 | 3.4 |
| WF14 | *Phocarctos hookeri* | sea lion | humerus |  |  | 11.7 | 17.5 | 33.4 | -12.7 | 3.3 |
| WF15 | *Phocarctos hookeri* | sea lion | humerus |  |  | 12.5 | 17.3 | 36.7 | -12.7 | 3.4 |
| WF16 | *Phocarctos hookeri (subadult)* | sea lion | humerus |  |  | 12.4 | 17.8 | 35.6 | -12.7 | 3.3 |
| WF17 | *Phocarctos hookeri* | sea lion | humerus |  |  | 11.9 | 18.2 | 34.5 | -12.3 | 3.4 |
| WF18 | *Phocarctos hookeri* | sea lion | humerus |  |  | 11.5 | 17.7 | 33.7 | -12.3 | 3.4 |
| WF19 | *Phocarctos hookeri* | sea lion | humerus |  |  | 9.8 | 17.5 | 28.8 | -12.5 | 3.4 |
| WF20 | *Emeus crassus* | Eastern Moa | femur |  |  | 8.9 | 4.4 | 26.6 | -24.0 | 3.5 |
| WF21 | *Emeus crassus* | Eastern Moa | tibiotarsus |  |  | 9.3 | 3.0 | 27.7 | -24.2 | 3.5 |
| WF22 | *Emeus crassus* | Eastern Moa | femur |  |  | 9.7 | 6.1 | 30.3 | -25.7 | 3.6 |
| WF23 | *Emeus crassus* | Eastern Moa | tibiotarsus |  |  | 12.3 | 6.3 | 36.6 | -23.8 | 3.5 |
| WF24 | *Emeus crassus* | Eastern Moa | tibiotarsus |  |  | 9.5 | 4.5 | 28.8 | -25.4 | 3.5 |
| WF25 | *Emeus crassus* | Eastern Moa | tibiotarsus |  |  | 11.8 | 5.3 | 35.2 | -26.0 | 3.5 |
| WF26 | *Anomalopteryx didiformis* | Bush Moa | tarsometatarsus |  |  | 11.6 | 7.2 | 34.3 | -21.9 | 3.5 |
| WF27 | *Anomalopteryx didiformis* | Bush Moa | femur |  |  | 12.2 | 2.8 | 36.4 | -23.4 | 3.5 |
| WF28 | *Anomalopteryx didiformis* | Bush Moa | tibiotarsus |  |  | 12.4 | 4.5 | 35.1 | -24.3 | 3.3 |
| WF29 | *Anomalopteryx didiformis* | Bush Moa | tibiotarsus |  |  | 10.4 | 8.2 | 30.3 | -21.7 | 3.4 |
| WF30 | *Euryapteryx curtis* | Coastal Moa | tarsometatarsus |  |  | 12.2 | 5.3 | 34.8 | -25.8 | 3.3 |
| WF31 | *Euryapteryx curtis* | Coastal Moa | femur |  |  | 10.4 | 7.4 | 32.1 | -24.1 | 3.6 |
| WF32 | *Euryapteryx curtis* | Coastal Moa | femur |  |  | 8.3 | 4.5 | 25.6 | -24.9 | 3.6 |
| WF33 | *Euryapteryx curtis* | Coastal Moa | tarsometatarsus |  |  | 11.0 | 5.8 | 33.1 | -25.1 | 3.5 |
| WF34 | *Dinornis robustus* | SI Giant Moa | tibiotarsus |  |  | 10.5 | 8.7 | 32.0 | -23.8 | 3.5 |
| **WF35** | ***Dinornis robustus*** | **SI Giant Moa** | **femur** |  |  | **7.6** | **6.7** | **24.0** | **-24.0** | **3.7** |
| WF36 | *Dinornis robustus* | SI Giant Moa | tibiotarsus |  |  | 9.5 | 6.2 | 29.0 | -25.0 | 3.6 |
| **WF37** | ***Dinornis robustus*** | **SI Giant Moa** | **femur** |  |  | **7.3** | **6.7** | **23.7** | **-23.7** | **3.8** |
| WB38 | *Hemiphaga novaeseelandiae* | NZ pigeon | humerus |  |  | 15.2 | 2.5 | 43.6 | -20.8 | 3.3 |
| *WB39* | *Hemiphaga novaeseelandiae* | *NZ pigeon* | *humerus* |  |  | *15.7* | *11.2* | *44.7* | *-18.3* | *3.3* |
| WB40 | *Hemiphaga novaeseelandiae* | NZ pigeon | coracoid |  |  | 15.5 | 3.0 | 44.1 | -21.0 | 3.3 |
| WB41 | *Hemiphaga novaeseelandiae* | NZ pigeon | ulna |  |  | 14.8 | 1.9 | 42.2 | -20.5 | 3.3 |
| WB42 | *Hemiphaga novaeseelandiae* | NZ pigeon | radius |  |  | 16.5 | 2.9 | 47.0 | -21.2 | 3.3 |
| WB43 | *Stictocarbo punctatus* | Spotted Shag | humerus |  |  | 15.6 | 16.4 | 44.6 | -12.3 | 3.3 |
| WB44.1 | *Stictocarbo punctatus* | Spotted Shag | humerus |  |  | 16.4 | 12.3 | 45.9 | -12.0 | 3.3 |
| WB44.2 | *Stictocarbo punctatus* | Spotted Shag | humerus |  |  | 16.1 | 12.2 | 45.1 | -12.1 | 3.3 |
| WB45 | *Stictocarbo punctatus* | Spotted Shag | humerus |  |  | 15.8 | 16.4 | 44.6 | -12.8 | 3.3 |
| WB46 | *Stictocarbo punctatus* | Spotted Shag | humerus |  |  | 15.3 | 16.8 | 44.1 | -13.5 | 3.4 |
| WB47 | *Stictocarbo punctatus* | Spotted Shag | humerus |  |  | 15.6 | 16.6 | 45.1 | -12.8 | 3.4 |
| WB48 | *Cygnus atratus* | Black Swan | humerus |  |  | 16.0 | 7.2 | 45.4 | -12.3 | 3.3 |
| WB49 | *Cygnus atratus* | Black Swan | humerus |  |  | 15.3 | 10.5 | 43.3 | -11.7 | 3.3 |
| WB50 | *Cygnus atratus* | Black Swan | humerus |  |  | 15.8 | 6.9 | 44.0 | -10.9 | 3.2 |
| WB51 | *Cygnus atratus* | Black Swan | humerus |  |  | 15.5 | 6.4 | 43.6 | -13.6 | 3.3 |
| WB52 | *Cygnus atratus* | Black Swan | humerus |  |  | 15.8 | 10.0 | 43.7 | -11.4 | 3.2 |
| WB53 | *Anas superciliosa* | Pacific Black Duck | humerus |  |  | 15.9 | 8.7 | 44.9 | -14.9 | 3.3 |
| WB54 | *Anas superciliosa* | Pacific Black Duck | scapula |  |  | 15.8 | 5.9 | 45.0 | -15.2 | 3.3 |
| WB55 | *Anas superciliosa* | Pacific Black Duck | carpometacarpus |  |  | 16.2 | 8.3 | 45.2 | -11.7 | 3.2 |
| WB56 | *Anas superciliosa* | Pacific Black Duck | carpometacarpus |  |  | 15.9 | 8.7 | 45.2 | -19.5 | 3.3 |
| WB57 | *Anas superciliosa* | Pacific Black Duck | humerus |  |  | 15.8 | 10.0 | 45.1 | -20.4 | 3.3 |
| WB58 | *Tadorna variegata* | Paradise Shelduck | coracoid |  |  | 15.8 | 7.0 | 44.7 | -17.2 | 3.3 |
| WB59 | *Tadorna variegata* | Paradise Shelduck | ulna |  |  | 16.0 | 6.9 | 47.2 | -12.5 | 3.4 |
| WB60 | *Tadorna variegata* | Paradise Shelduck | fercula |  |  | 15.5 | 4.2 | 44.6 | -21.4 | 3.4 |
| WB61 | *Tadorna variegata* | Paradise Shelduck | fibula |  |  | 16.6 | 10.5 | 47.1 | -15.4 | 3.3 |
| *WB62* | *Tadorna variegata* | *Paradise Shelduck* | *humerus* |  |  | *15.5* | *16.9* | *44.5* | *-13.0* | *3.3* |
| WB64 | *Aythya novaeseelandiae* | Black Teal | coracoid |  |  | 15.6 | 6.8 | 44.2 | -14.5 | 3.3 |
| WB65 | *Aythya novaeseelandiae* | Black Teal | tibiotarsus |  |  | 16.9 | 9.5 | 47.4 | -12.8 | 3.3 |
| WB66 | *Aythya novaeseelandiae* | Black Teal | tibiotarsus |  |  | 15.6 | 9.5 | 44.9 | -12.7 | 3.4 |
| *WB67* | *Aythya novaeseelandiae* | *Black Teal* | *scapula* |  |  | *15.0* | *16.2* | *43.0* | *-12.9* | *3.3* |
| WB68 | *Anas chlorotis* | Brown teal | radius |  |  | 16.2 | 10.0 | 46.2 | -21.7 | 3.3 |
| WB69 | *Fulica prisca* | NZ Coot (extinct rail) | tarsometatarsus |  |  | 15.2 | 11.2 | 44.5 | -13.9 | 3.4 |
| WB70 | *Phalacrocorax melanoleucos* | Little Pied Cormorant | humerus |  |  | 16.5 | 4.4 | 47.0 | -19.0 | 3.3 |
| WB71 | *Leucocarbo carunculatus* | King Shag | crania |  |  | 16.3 | 6.6 | 45.7 | -17.6 | 3.3 |
| WB72 | *Larus dominicanus* | Kelp Gull | tarsometatarsus |  |  | 14.8 | 10.8 | 42.9 | -14.1 | 3.4 |
| WB73 | *Larus bulleri* | Black-billed Gull | coracoid |  |  | 13.9 | 16.2 | 41.0 | -14.7 | 3.5 |
| WB74 | *Larus bulleri* | Black-billed Gull | carpometacarpus |  |  | 16.1 | 18.5 | 45.0 | -15.1 | 3.3 |
| WB75 | *Eudyptula minor* | Little Penguin | radius |  |  | 14.7 | 17.0 | 42.6 | -13.4 | 3.4 |
| WB76 | *Arripis trutta* | Kahawai fish | premaxilla |  |  | 15.3 | 13.5 | 42.6 | -12.9 | 3.2 |
| WB77 | *Arripis trutta* | Kahawai fish | pharyngeal |  |  | 16.2 | 13.5 | 43.7 | -11.6 | 3.2 |
| WB78 | *Arripis trutta* | Kahawai fish | dentary |  |  | 15.4 | 13.7 | 43.2 | -12.8 | 3.3 |
| WB79 | *Arripis trutta* | Kahawai fish | quadrate |  |  | 15.3 | 13.2 | 43.3 | -13.0 | 3.3 |
| WB80 | *Arripis trutta* | Kahawai fish | hyomandibular |  |  | 14.9 | 13.1 | 42.5 | -12.2 | 3.3 |
| WB81 | *Arripis trutta* | Kahawai fish | dentary |  |  | 15.4 | 15.0 | 44.2 | -12.0 | 3.4 |
| WB82 | *Thyrsites atun* | Barracouta | dentary |  |  | 15.8 | 14.2 | 44.6 | -12.9 | 3.3 |
| WB83 | *Rattus exulans* | Pacific rat | tibia |  |  | 13.9 | 17.4 | 41.1 | -16.1 | 3.5 |
| WB84 | *Rattus exulans* | Pacific rat | femur |  |  | 13.8 | 17.4 | 41.4 | -20.3 | 3.5 |
| WB85 | *Rattus exulans* | Pacific rat | pelvis |  |  | 16.0 | 15.5 | 45.2 | -17.2 | 3.3 |
| WB86 | *Rattus exulans* | Pacific rat | humerus |  |  | 13.9 | 15.2 | 40.2 | -17.3 | 3.4 |
| WB87 | *Rattus exulans* | Pacific rat | femur |  |  | 15.3 | 15.4 | 44.3 | -20.9 | 3.4 |
| WB88 | *Rattus exulans* | Pacific rat | tibia |  |  | 15.1 | 14.9 | 42.8 | -17.2 | 3.3 |

Legend: Italicized samples delineate those suspected of misidentification because of anomalously high δ^15^N values and were therefore not included in the descriptive statistics in the text.

Bolded samples displayed C:N ratios outside 2.9-3.6
