# Supplementary material for: The First New Zealanders: Patterns of Diet and Mobility Revealed through Isotope Analysis
Source: PLoS One. 2013 May 15;8(5):e64580. doi: 10.1371/journal.pone.0064580 (PMC3654917; doi:10.1371/journal.pone.0064580)
Supplement: Table S2 — Demographic information, burial specifics, and isotope data for the Wairau Bar humans. (DOCX) [file pone.0064580.s004.docx]

Table S2. Demographic information, burial specifics, and isotope data for the Wairau Bar humans.

| Burial | Group | Age^a^ | Sex^b^ | Position^c^ | Moa egg^d^ | Bone sample | Tooth^e^ | ^87^Sr/^86^Sr | ±^87^Sr/^86^Sr (2 S.E.) | %N | δ^15^N (‰) | %C | δ^13^C (‰) | C:N |
| --- | --- | --- | --- | --- | --- | --- | --- | --- | --- | --- | --- | --- | --- | --- |
| 1 | 1 | YA-MA | F | Prone | P |  | 16 | 0.707741 | 0.000014 |  |  |  |  |  |
| 2.1 | 1 | YA | M | Prone | P | femoral shaft | 26 | 0.707297 | 0.000012 | 9.9 | 13.9 | 29.3 | -18.3 | 3.4 |
| 2.2 | 1 | YA | M? | No info | P | hand phalanx |  |  |  | 9.1 | 16.8 | 27.9 | -17.2 | 3.6 |
| 3 | 1 | MA | M | Prone | P | femoral shaft |  |  |  | 9.1 | 14.9 | 27.6 | -18.2 | 3.5 |
| 4 | 1 | MA-OA | M | Prone | P | femoral shaft | 16 | 0.706510 | 0.000009 | 8.7 | 14.6 | 26.2 | -17.8 | 3.5 |
| 5 | 1 | MA-OA | M? | Prone | P | fibular shaft | 28 | 0.707680 | 0.000014 | 12.2 | 14.2 | 36.4 | -18.0 | 3.5 |
| 6 | 1 | YA-MA | M | Prone | P | femoral shaft | 16 | 0.707990 | 0.000015 | 8.9 | 13.1 | 22.6 | -17.1 | 3.0 |
| 7 | 1 | MA | UK | Secondary | P | vertebrae | PM | 0.708032 | 0.000014 | 14.3 | 14.2 | 42.5 | -17.5 | 3.5 |
| 8 | 2 | OA | F | Left side | NP | fibular shaft | 16 | 0.708832 | 0.000012 | 13.4 | 18.9 | 39.2 | -14.7 | 3.4 |
| 9 | 2 | UK | UK | Right side | NP | tibial shaft |  |  |  | 13.0 | 17.2 | 40.3 | -17.5 | 3.6 |
| 11.1 | 2 | UK | F | No info | NP | femoral shaft |  |  |  | 12.9 | 19.3 | 38.0 | -14.2 | 3.4 |
| 12 | 3 | YA-MA | F | Right side | NP | crania | 16 | 0.708279 | 0.000015 | 14.2 | 15.8 | 41.4 | -19.1 | 3.4 |
| 13 | 3 | YA | F | Right side | NP | vertebrae | 36 | 0.708931 | 0.000012 | 17.7 | 15.8 | 52.7 | -17.7 | 3.5 |
| 14 |  | OA | M? | Prone | P |  | 47 | 0.707188 | 0.000012 |  |  |  |  |  |
| *15* | *3* | *OA* | *F?* | *Flexed* | *NP* | *tibial shaft* |  |  |  | *7.9* | *14.5* | *25.0* | *-18.0* | *3.7* |
| 16.1 | 3 | YA | F | Secondary | NP | femoral shaft | 36 | 0.708185 | 0.000013 | 10.5 | 20.1 | 32.1 | -15.7 | 3.6 |
| *16.2* | *3* | *OA* | *F* | *Commingled* | *NP* | *crania* |  |  |  | *8.0* | *17.6* | *25.7* | *-16.4* | *3.8* |
| *17* | *3* | *YA* | *F* | *Supine* | *NP* | *crania* | *26* | *0.708664* | *0.000012* | *11.4* | *15.8* | *41.1* | *-17.7* | *4.2* |
| *18* | *3* | *YA-MA* | *F* | *Secondary* | *NP* | *femoral shaft* | *46* | *0.708787* | *0.000010* | *12.6* | *19.3* | *43.1* | *-15.9* | *4.0* |
| *19* | *3* | *YA* | *M?* | *Prone* | *NP* | *crania* |  |  |  | *10.3* | *13.9* | *33.9* | *-19.8* | *3.8* |
| 20 | 3 | UK | F? | Right side | P | fibular shaft |  |  |  | 9.5 | 14.5 | 29.2 | -18.8 | 3.6 |
| 21 |  | MA | F | Supine | NP |  | 38 | 0.708386 | 0.000011 |  |  |  |  |  |
| 22.1 |  | YA | F | Supine | NP |  | 16 | 0.709382 | 0.000010 |  |  |  |  |  |
| *22.2* | *3* | *OA* | *F* | *Commingled* | *NP* | *mandible* |  |  |  | *5.0* | *17.2* | *17.8* | *-18.0* | *4.2* |
| *24* | *3* | *OA?* | *M* | *Supine* | *NP* | *mandible* |  |  |  | *3.5* | *14.1* | *12.1* | *-17.4* | *4.0* |
| 25 | 3 | YA | M | Left side | NP | crania | 16 | 0.709208 | 0.000013 | 11.0 | 16.6 | 33.4 | -17.4 | 3.6 |
| 26 | 3 | YA | UK | Left side | NP | crania | 46 | 0.708812 | 0.000011 | 16.3 | 12.1 | 48.0 | -19.8 | 3.4 |
| 27 | 3 | YA | UK | No info | NP | crania | 26 | 0.708406 | 0.000013 | 15.9 | 15.1 | 47.6 | -17.9 | 3.5 |
| *28* | *3* | *UK* | *F* | *Crouched* | *NP* | *mandible* |  |  |  | *8.0* | *15.9* | *25.2* | *-18.8* | *3.7* |
| 29 | 3 | OA | M | Supine | NP | crania |  |  |  | 12.8 | 15.9 | 36.1 | -18.5 | 3.3 |
| 30 | 3 | OA | F | Prone | P | crania | 38 | 0.708557 | 0.000013 | 10.7 | 15.5 | 32.6 | -17.6 | 3.5 |
| 31 | 3 | YA | F | Prone | P | crania | 16 | 0.708423 | 0.000009 | 11.5 | 15.9 | 31.7 | -19.4 | 3.2 |
| *33* | *3* | *UK* | *UK* | *Left side* | *NP* | *crania* | *17* | *0.707652* | *0.000013* | *7.5* | *16.7* | *25.6* | *-18.0* | *4.0* |
| 35 | 3 | YA | M | Prone | NP | rib | 37 | 0.709139 | 0.000013 | 10.9 | 13.9 | 32.8 | -21.2 | 3.5 |
| 36 | 3 | YA | M | No info | P | crania |  |  |  | 10.3 | 15.2 | 31.6 | -20.2 | 3.6 |
| *37* | *3* | *MA-OA* | *F* | *Supine* | *NP* | *crania* | *27* | *0.708440* | *0.000012* | *5.3* | *14.3* | *17.9* | *-18.5* | *3.9* |
| 40 | 3 | OA | M | No info | NP | crania |  |  |  | 10.7 | 18.4 | 31.7 | -15.3 | 3.5 |
| 41.1 | 3 | OA | F | Prone | P | crania | 16 | 0.708659 | 0.000014 | 14.0 | 17.8 | 40.7 | -15.7 | 3.4 |
| 42 | 3 | OA | F | Right side | NP | crania |  |  |  | 10.5 | 17.1 | 31.7 | -16.3 | 3.5 |
| 43 | 3 | OA | M? | No info | NP | crania |  |  |  | 12.8 | 15.9 | 38.1 | -18.0 | 3.5 |
| 44 | 3 | UK | UK | Flexed | NP | long bone shaft |  |  |  | 15.1 | 17.7 | 44.2 | -15.8 | 3.4 |
| *491* | *3* | *UK* | *UK* | *No info* | *NP* | *tibial shaft* |  |  |  | *2.8* | *16.4* | *14.6* | *-21.4* | *6.1* |

Legend: ^a^YA=Young adult (17-34 years), MA=Mid adult (35-49 years), OA=Old adult (50+) and UK=Adult with unknown age

^b^ M=Male, F=Female and UK=Adult of unknown sex

^c^ Burial position

^d^ Presence of moa egg: P=present and NP=not present

^e^ Tooth type sampled, PM=premolar

Italicized samples displayed C:N ratios outside 2.9-3.6
